# One Health Index Calculator for India Using Empirical Methods for Policy Stewardship: Development and Usability Study

**DOI:** 10.2196/65039

**Published:** 2025-06-25

**Authors:** Saveetha Meganathan, Arpit Katiyar, Esha Srivastava, Rakesh Kumar Mishra

**Affiliations:** 1 Tata Institute for Genetics and Society, Bengaluru, 560065, India, 91 9441902188

**Keywords:** one health, one health index calculator, fuzzy extent analysis, modified entropy-based weightage method, policy stewardship, India, low and middle income countries, LMIC, Index, indicator, secondary data collection

## Abstract

**Background:**

*One Health* is a collaborative approach that can be used to evaluate and enhance the fields of human, animal, and environmental health and to emphasize their sectoral interconnectedness. Empirical evaluation of the *One Health* performance of a country in the form of an index, provides direction for actionable interventions such as targeted funding, prioritized resource allocation, rigorous data management, and evidence-based policy decisions. These efforts, along with public engagement and awareness on disease management; environmental degradation, and preparedness toward disease outbreaks, contribute to strengthening global health security. Thus, developing a One Health Index (OHI) calculator for India is a significant step toward evidence-based *One Health* governance in the context of low-and middle-income countries.

**Objective:**

This study aimed to (1) develop a OHI Calculator for India using efficient and user-friendly weighting methods and demonstrate the calculation of the OHI; (2) develop India-specific datasets through secondary data collection from reliable data sources; and (3) determine data gaps for policy stewardship.

**Methods:**

We proposed a OHI calculator to measure the OHI from an Indian context by adopting the Global One Health Index framework that comprises 3 categories: 13 key indicators, 57 indicators, and 216 subindicators. Secondary data collection was conducted to create a dataset for specific to India from reliable sources. For measuring OHI, we demonstrated two mathematical weighting methods: an efficient expert-based rating using fuzzy extent analysis and a modified entropy-based weightage method.

**Results:**

We demonstrate the step-by-step OHI calculation by determining indicator scores using both fuzzy extent analysis and modified entropy-based weightage method. Through secondary data collection an India-specific dataset was created using reliable sources. For the datasets from India, data for 156/216 subindicators were available, while that for the remaining 60 indicators were unavailable. Further, a pilot correlation analysis was performed between 20 indicator scores and relevant budget allocations for the years 2022‐2023, 2023‐2024, and 2024‐2025. It was found that increases in the budget allocation across consecutive years improved indicator scores or better performance and vice versa.

**Conclusions:**

The demonstrated OHI calculator has the potential to serve as a governance tool while promoting data transparency and ethical data management. There is a need for a collaborative data federation approach to resolve data gaps, including incomplete, missing, or unavailable data. Further, the correlation analysis between budgetary allocation and performance of indicators provides empirical evidence for policymakers to improve intersectoral communication, multistakeholder engagement, concerted interventions, and informed policy decisions for resource allocation.

## Introduction

*One Health* is a participatory, collaborative approach to enhancing the health of people, animals, and ecosystems over time. It recognizes the interdependence of the health of humans, domestic and wild animals, plants, and the larger environment. While health, food sources, water, energy, and the environment are broad topics with sector-specific concerns, cross-sectoral and cross-disciplinary collaboration helps protect human health, address health challenges such as the emergence of infectious diseases and antimicrobial resistance, ensure food safety, and promote the health and integrity of ecosystems. The *One Health* approach has the potential to address the complete spectrum of disease control, from prevention to detection, readiness, response, and management, while also contributing to global health security by considering the multisectoral interconnectedness and their mutual impact [1].

The term ’One Health’ evolved during the outbreaks of SARS in 2003‐2004 and H5N1 influenza (ie, bird flu), highlighting the interconnectedness of human, animal, and environmental health. The ’Manhattan Principles’ underscored this link, recognizing the need for collaborative approaches in global disease prevention [2]. These outbreaks emphasized the risks posed by unknown pathogens from wildlife, which led to the development of more effective alert and response systems. Global cooperation involving United Nations, World Health Organization, Food and Agriculture Organization, World Organization for Animal Health, United Nations Children’s Fund, and the World Bank addressed the H5N1 outbreak, with the International Ministerial Conference on Avian and Pandemic Influenza playing a key role [3]. The primary drivers of the emergence of novel zoonotic infectious diseases include human activities, changes to ecosystems, land use, agriculture intensification, urbanization, international travel, and trade over the past three decades. These diseases, predominantly originating in wildlife, pose significant public health risks. The *One Health* approach is pivotal for the prevention, monitoring, and surveillance of zoonotic and emerging infectious diseases, sustaining food security, and combating antimicrobial resistance, all of which affect human, animal, and environmental health. Subsequently, collaborative monitoring systems have become essential for effectively managing pandemics and outbreaks, given the multitude of health crises in the last decade [3].

Developing a *One Health* Index (OHI) framework can contribute to the implementation of the *One Health* approach by focusing on intersectoral collaborations and the corresponding datasets. The value of such an index extends beyond compiling data; it has the potential to revolutionize our understanding, management, and response to the complex web of factors that influence global health. A OHI simplifies health data, making it accessible to diverse stakeholders including government functionaries, policymakers, health care providers and the public [4]. It condenses a huge volume of information into a single quantifiable value. Government and health care organizations can use these health indices to make informed policy decisions regarding the allocation of resources and implementation of public health interventions [5]. Further, these indices facilitate the monitoring of health trends in a given region or country, enabling the assessment of the effectiveness of public health campaigns and improvements in health care systems and policy frameworks [6,7].

In this context, the Global One Health Index (GOHI) serves as an empirical tool for the systematic assessment of *One Health* scores for more than 200 countries and territories worldwide [8]. The GOHI framework consists of three major categories—extrinsic driver index, core driver index, and intrinsic driver index—which are further categorized into 13 key-indicators, 57 indicators, and 216 subindicators. This structure makes it possible to quantify the *One Health* Index for a country by mapping the multisectoral variables that contribute to the well-being of humans, animals, and the environment and their mutual interactions [8]. Additionally, the GOHI framework accounts for the recruitment of 29 domain experts to attribute weightage to the indicators based on their sectoral experience, further demonstrating the need for cross-sectoral communication and concerted efforts to achieve better *One Health* outcomes for a country [8].

In this study, we proposed to adapt and contextualize the *One Health* Index calculator for India based on the GOHI framework. We demonstrated the calculation of indicator scores using two mathematical weighting methods: (1) the efficient expert-based fuzzy extent analysis (FEA) method that requires recruitment of domain experts, and (2) the modified entropy-based weightage method (MEWM), which replaces expert-based weightage calculation with mathematical formula-based calculations. A country-specific database for India was developed through secondary data collection. The two weighting methods—FEA and MEWM—were demonstrated using the India-specific dataset to generate indicator scores, subject to data availability. In addition, we correlated sectoral budget allocations over the last two financial years with corresponding sectoral indicator scores. This analysis serves as an actionable pointer for policy makers, federal and state governments to support decision-making toward budgetary allocations.

## Methods

The OHI calculator for India is aimed to serve as a public health tool that addresses the need for multisectoral collaboration for data access and understanding the impact of sectoral performance, thereby improving the countrywide *One Health* performance. This calculator consists of the following steps:

### Indicator Selection

To demonstrate the calculation of the OHI for India, we adopted three categories: 13 key indicators, 57 indicators, and 216 subindicators from the GOHI [8] (Multimedia Appendix 1).

### Database Building

For the demonstration of the OHI calculator for India, national-level data collection was conducted using secondary data sources such as the Press Information Bureau (Government of India), Department of Agriculture and Farmers Welfare (Government of India), World Bank, Food and Agriculture Organization of the United Nations, Yale Environmental Performance Index (Yale University), Our World in Data, The Global Economy, Statista, IndiaStat, Knoema, and other relevant databases (Multimedia Appendix 2). Additionally, to correlate the sectoral budgets over the last two financial years with their respective indicator scores, the budget datasets were obtained from the Indian Union Budget documents for the financial years 2022‐2023, 2023‐2024, and 2024‐2025 (Multimedia Appendix 3).

### Weight Determination

A sample proforma was developed to obtain expert ratings for various subindicators, indicators, key indicators, and categories. This proforma can be self-administered via email or used for in-person interviews, thereby providing efficient data collection from experts. It also allows the experts to provide additional information such as variables, data sources, and case studies (Multimedia Appendix 4).

### Procedure to Calculate Expert Weights

There are two mathematical weightage methods for calculating the indicator scores: (1) fuzzy extent analysis (FEA)–an efficient expert-based method and (2) Modified Entropy-Based Weightage Method (MEWM)–a data-driven method.

### Fuzzy Extent Analysis (FEA)

The efficient expert-based FEA rating method used to calculate the indicator scores requires consultations with experts from diverse *One Health* sectors for ascertaining the priority or the weightage of different subindicators and indicators. The FEA is a multicriteria decision-making method that integrates both qualitative and quantitative approaches [9]. Data collection for expert-based rating can be conducted using the provided proforma. A pairwise comparison was used for developing the proforma, that allowed the experts to rate the metrics [10]. It is an effective way to gather opinions from many experienced experts, especially for complex decision-making problems involving multiple risks. The following is an example of how expert-based ratings are converted into weights. A pair of subindicators within an experts’ domain will be provided to them for pairwise comparison using linguistic ratings, which are then translated into an existing numerical scale of relative importance [10]. Similarly, all possible combinations of subindicators will be provided to the expert for comparisons. The linguistic ratings obtained from an expert, such as equally important, moderately important, strongly important, and extremely important will be converted into numerical ratings. These numerical ratings are further converted into comparison matrix. Further, the consistency of the responses by the experts will be checked using the consistency ratio. After ascertaining the consistency of the responses, the next step will be “fuzzification” (ie, conversion of the numerical ratings to fuzzy numbers). To do this, we will use triangular fuzzy numbers (defined as a generalization of real numbers representing a set of possible values with weights, or membership functions) to calculate the weightage of different subindicators and indicators. Similarly, weights for the key indicators and categories may be obtained using this efficient expert-based weightage mechanism, or through a format similar to panel discussions which can be conducted to obtain the weights from the domain experts.

### Steps involved for OHI calculation using FEA

#### Step 1

1. Consult with domain experts from diverse sectors relevant to *One Health*.
2. Based on the systematic implementation of the semistructured interviews and a modified Delphi method, pairwise comparisons are made on the importance of each pair of parameters.
3. Given a hierarchy with n parameters, there will be n(n – 1)/2 pairwise comparisons.
4. The comparisons are rated linguistically.
5. Linguistic variables are converted to numerical ratings using scale of relative importance [11].

#### Step 2

A comparison matrix will be established for each expert using the ratings from the proforma. The comparison matrix for the $k^{th}$expert is given by:

${\tilde{A}}^{k}=[a_{ij}]$ represented as:

${\tilde{A}}^{K}$=$\left(\begin{array}{ccc} 1 & \cdots & {\tilde{a}}_{1n}\\ \vdots & \ddots & \vdots \\ {\tilde{a}}_{n1} & \cdots & 1 \end{array}\right)$

The reciprocal matrix is denoted by ${\left[{\tilde{A}}^{K}\right]}^{-1}$.

The reciprocity properties are given by:


$$a_{ij}{\;}^{-1}=\frac{1}{a_{ij}},\forall \;i,j=1,2,3,...n$$

#### Step 3

It crucial to maintain consistency for expert-based weightage process to derive the indicator scores. To achieve this, a Consistency Index (CI) was calculated to evaluate the consistency of the comparison matrix [10]:


$$CI=\frac{\lambda_{max}-n}{n-1}$$

where $\lambda_{max}$ is the maximum eigen value and *n* is the dimension of the comparison matrix [9].

The consistency ratio is given by:


$$CR=\frac{CI}{RI}$$

where, RI is the random consistency index which depends on *n* [10].
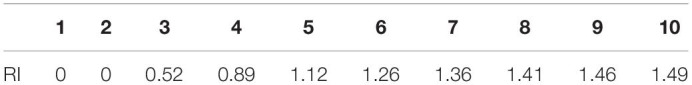


If CR <0.1, the judgements are considered consistent [12].

#### Step 4

1. Convert the ratings in the comparison matrix to triangular fuzzy number.
2. A fuzzy number serves as a tool to express values that are uncertain or imprecise particularly in the context of fuzzy set theory.
3. Unlike a value, it accommodates varying degrees of belongingness enabling it to account for the ambiguity often found in practical scenarios.
4. Fuzzy sets can compensate for the inconsistency and imprecisions in human judgements rather than random or stochastic ones.
5. Fuzzy numbers are depicted by a set of possible values having their own membership function ranging from 0 to 1.
6. A triangular fuzzy number is represented by [floor value, average value, ceiling value] ie, (l,m,u) with the member function as:

$$\mu_{M}\left(x\right)=\left\{ \begin{array}{c} \frac{x}{m-l}-\frac{l}{m-l},x∊\left[l,m\right]\\ \frac{x}{m-u}-\frac{u}{m-u},x∊\left[l,m\right]\\ 0,otherwise \end{array}\right.$$

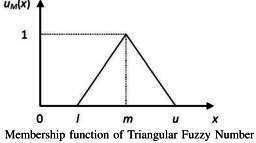
[13].

#### Step 5

To calculate weights from the comparison matrix, FEA is applied [13].

In extent analysis, let $X=\left\{x_{1,}x_{2,...,}x_{n}\right\}$ be an object set and $G=\left\{g_{1},g_{2},...,g_{n}\right\}$ a goal set. For each object, extent analysis is performed for each goal $g_{i}$.

The value of fuzzy synthetic extent is given by:


$$S_{i}=\sum_{j=1}^{m}M_{g_{i}}{\;}^{j}⊗{\left[\sum_{i=1}^{n}\sum_{j=1}^{m}M_{g_{i}}{\;}^{j}\right]}^{-1}$$

where,


$$\sum_{j=1}^{m}M_{g_{i}}{\;}^{j}=\left(\sum_{j=1}^{m}l_{j},\sum_{j=1}^{m}m_{j},\sum_{j=1}^{m}u_{j}\right)$$

We can calculate the ratings in the triangular fuzzy number format as shown above leading to a weight derived from these triangular weights.

A pairwise comparison of fuzzy weights needs to be performed and the computation of the degree of possibility of them being greater than the fuzzy weight will be obtained. The minimum of these possibilities is used as the overall score for each criterion *i*.

To compute the value of the fuzzy synthetic extent, $S_{i}$ for the $i^{th}$ object is as follows:


$$S_{i}=\sum_{j=1}^{m}M_{i}{\;}^{j}⊗{\left[\sum_{i=1}^{n}\sum_{j=1}^{m}M_{i}{\;}^{j}\right]}^{-1}$$

where,


$$\sum_{i=1}^{m}M_{i}{\;}^{j}=\left(\sum_{j=1}^{m}l_{i}{\;}^{j},\sum_{j=1}^{m}m_{i}{\;}^{j},\sum_{j=1}^{m}n_{i}{\;}^{j}\right)$$

$${\left[\sum_{i=1}^{n}\sum_{j=1}^{m}M_{i}{\;}^{j}\right]}^{-1}=\left(\frac{1}{\sum_{i=1}^{n}\sum_{j=1}^{m}u_{i}{\;}^{j}}\;,\;\frac{1}{\sum_{i=1}^{n}\sum_{j=1}^{n}m_{i}{\;}^{j}}\;,\;\frac{1}{\sum_{i=1}^{n}\sum_{j=1}^{n}l_{i}{\;}^{j}}\right)$$

#### Step 6

The fuzzy synthetic extent constitutes three values which will be averaged out to acquire a single value for the weights from them.

Weights = $\sum_{i=1}^{k}\tilde{W_{i}}/k$

#### Step 7

Normalize each weight by dividing the individual weights by the sum of all weights.


$$Normalized\;weight{\;}_{i}=W_{i}/\sum_{i=1}^{k}W_{i}$$

#### Step 8

If the ratings have been collected from multiple experts for the same indicator, either of these methods can be used to obtain optimized ratings:

##### Method 1

1. Calculate individual normalized weights using Steps 1-7 for every expert rating
2. Average the normalized weights to obtain optimized ratings for the indicators

##### Method 2

In continuation to Step 5, after calculating the fuzzy synthetic extent for every expert input individually,

1. Compute the degree of possibility of

$S_{2}\left(l_{2},m_{2},u_{2}\right)\geq S_{1}\left(l_{1},m_{1},u_{1}\right)$

  where degree of possibility between two fuzzy synthetic extents is defined as:

$V\left(S_{2}\geq S_{1}\right)=sup_{y\leq x}\left[min\left(\mu_{S_{2}}\left(y\right),\mu_{S_{1}}\left(x\right)\right)\right]$

$V\left(S_{2}\geq S_{1}\right)=hgt\left(S_{1}\cap S_{2}\right)=\mu_{S_{2}}\left(d\right)$

  where, $\mu_{S_{2}}\left(d\right)=\left\{ \begin{array}{l} \;1,\;\;\;\;if\;\;m_{2}\geq \;m_{1}\\ \;0,\;\;\;if\;\;l_{1}\geq \;u_{2}\\ \frac{l_{1}-u_{2}}{m_{2}-u_{2}},\;\;otherwise \end{array}\right.$
  and› d is the ordinate of the highest intersection point d between ${µ}_{S_{1}}$ and ${µ}_{S_{2}}$.

1. Compute the degree of possibility for a convex fuzzy number to be >k convex fuzzy numbers $S_{i}$(1, 2, …, k)

$\begin{array}{l} V\;[(S\geq S_{1},S_{2},...,S_{k})=V[(S\geq S_{1})\;and\\ (S\geq S_{2})\;...and\;(S\geq S_{k})]=min\;V\left(S\geq S_{i}\right),\;i=1,2,...,k \end{array}$

2. Compute the vector W’.

$W^{\prime }={\left(d^{\prime }\left(A_{1}\right),d^{\prime }\left(A_{2}\right),...,d^{\prime }(A_{k})\right)}^{T}$

  $d\prime \left(A_{i}\right)=\min V\left(S_{i}\;\geq \;S_{j}\right)$ Where
  for i=1, 2, …, k and j=1, 2, …, k and i ≠ j
  Normalized vector, $W={\left(d\left(A_{1}\right),d\left(A_{2}\right),\ldots ,d\left(A_{k}\right)\right)}^{T}$
  W is a nonfuzzy number calculated for each comparison matrix [14],
  Now, the weights have been calculated through FEA, thus using these weights and an appropriate weight accumulation formula, OHI can be calculated.

### Step 9

For the accumulation of the indicators and the weights, the following formula can be used:


$$S_{ij}=\left\{ \begin{array}{l} 0\\ \frac{X_{ij}-X_{worst,j}}{X_{best,j}-X_{worst,j}}\\ 100 \end{array}\right.*100$$

where $S_{ij}$ denotes the normalized score for the $j^{th}$ sub indicator of $i^{th}$ indicator; $X_{ij}$ denotes the original values for the $j^{th}$ sub indicator of $i^{th}$ indicator, $X_{best,j}$ denotes the best value for the $j^{th}$ sub indicator of the $i^{th}$ indicator, $X_{worst,j}$ denotes the worst value for the $j^{th}$ sub indicator of the $i^{th}$indicator. In cases where no data is available for the sub-indicators, substitute $S_{ij}$ with 0.

The weighted sum of the scores can be given by:

$Indicator\;Score_{ih}=\sum_{1_{h}}^{m_{h}}S_{ij_{h}}*W_{j_{h}},\sum_{1_{h}}^{m_{h}}W_{j_{h}}=1$,

where, m depicts the number of subindicators under the $h^{th}$ indicator, $j_{h}$ depicts the $j^{th}$subindicator under the $h^{th}$ indicator, $S_{{ij}_{h}}$ depicts the score of the ${j_{h}}^{th}$ sub indicator under $i^{th}$ indicator; $W_{j_{h}}$ depicts the weight of ${j_{h}}^{th}$ subindicator [8].

The procedure is repeated stage-wise across sub-indicators, indicators, key indicators, and categories to calculate the OHI.

### Modified Entropy Based Weightage Method (MEWM)

This approach requires the indicators values for OHI calculation. Weightage mechanism is backed by the entropy-based weightage method for the indicator value and is a preferred method due to the ease of calculation and does not require expert-based rating.

#### Step 1

First, normalization (a systematic process of organizing data in a database to make it more flexible and cohesive) of the values of the sub indicators will be done using the following formula,

$r_{ij}$=$r_{ij}=\frac{x_{ij}-x_{min,j}}{max\;x_{j}-\min x_{j}}$

where max $x_{j}$ and min $x_{j}$ are the maximum and minimum values among the alternatives for indicator *j* [15] .

#### Step 2

The entropy $E_{ij}$ of each subindicator *i* from the indicator *j*, the entropy $E_{ij}$ of each indicator was determined from the normalized values $r_{ij}$ as formulated:


$$E_{ij}=-\;\frac{f_{ij}*lnf_{ij}}{\ln n}\;\;\;\;\left(i=1,\ldots ,n,\;j=1,\ldots ,m\right)$$

Where $f_{ij}=\;r_{ij}/\sum_{i=1}^{n}r_{ij}$

For the cases where $f_{ij}$= 0or is not available, the entropy becomes not defined and, in those cases, substitute the entropy with 0.

#### Step 3

For the applicability of the method, the weights $w_{ij}$ are computed as defined below:

$w_{ij}=\frac{1-E_{ij}}{n-\sum_{i=1}^{n}E_{ij}}$ , $\sum_{i=1}^{n}w_{ij}=1$, (i=1,…,n)

#### Step 4

The above formula can be used at every step to calculate the relevant weights,


$$V_{ij}=w_{ij}*r_{ij}\;\;\;\;\;\;\;\left(i=1,\ldots ,n,\;\;j=1,\ldots ,m\right)$$

where $r_{ij}$ is the standardized value of the $i^{th}$ sub-indicator for the $j^{th}$ state and $w_{ij}$ being the weight calculated for the $i^{th}$ sub-indicator for the $j^{th}$ state; $v_{ij}$ is the weighted indicator value.


$$r_{i,\;j+1}=v_{j}=\sum_{i=1}^{n}v_{ij}$$

where $v_{ij}$ is the weighted indicator value for the $i^{th}$ subindicator for the $j^{th}$ state and $v_{j}$ is the index value of the indicator j using which, $r_{j}$ is calculated.

One can use this formula to arrive at the weighted value of the subindicators and then use those subindicator values to derive weights for the indicators and similarly repeating the process for all available data. These steps will transition stagewise in an agglomerative way from the scores of the sub indicators of a particular indicator to the OHI.

### Ethical Considerations

A formal ethical review was waived. This study was not submitted for review as the Tata Institute for Genetics and Society (TIGS) adheres to Indian Council for Medical Research ethical guidelines, which allow exemption for minimal-risk studies using publicly available data. Further, our manuscript is solely based on secondary data from publicly available databases and these were appropriately cited to maintain research integrity.

## Results

### Primary findings

**Figure 1.**
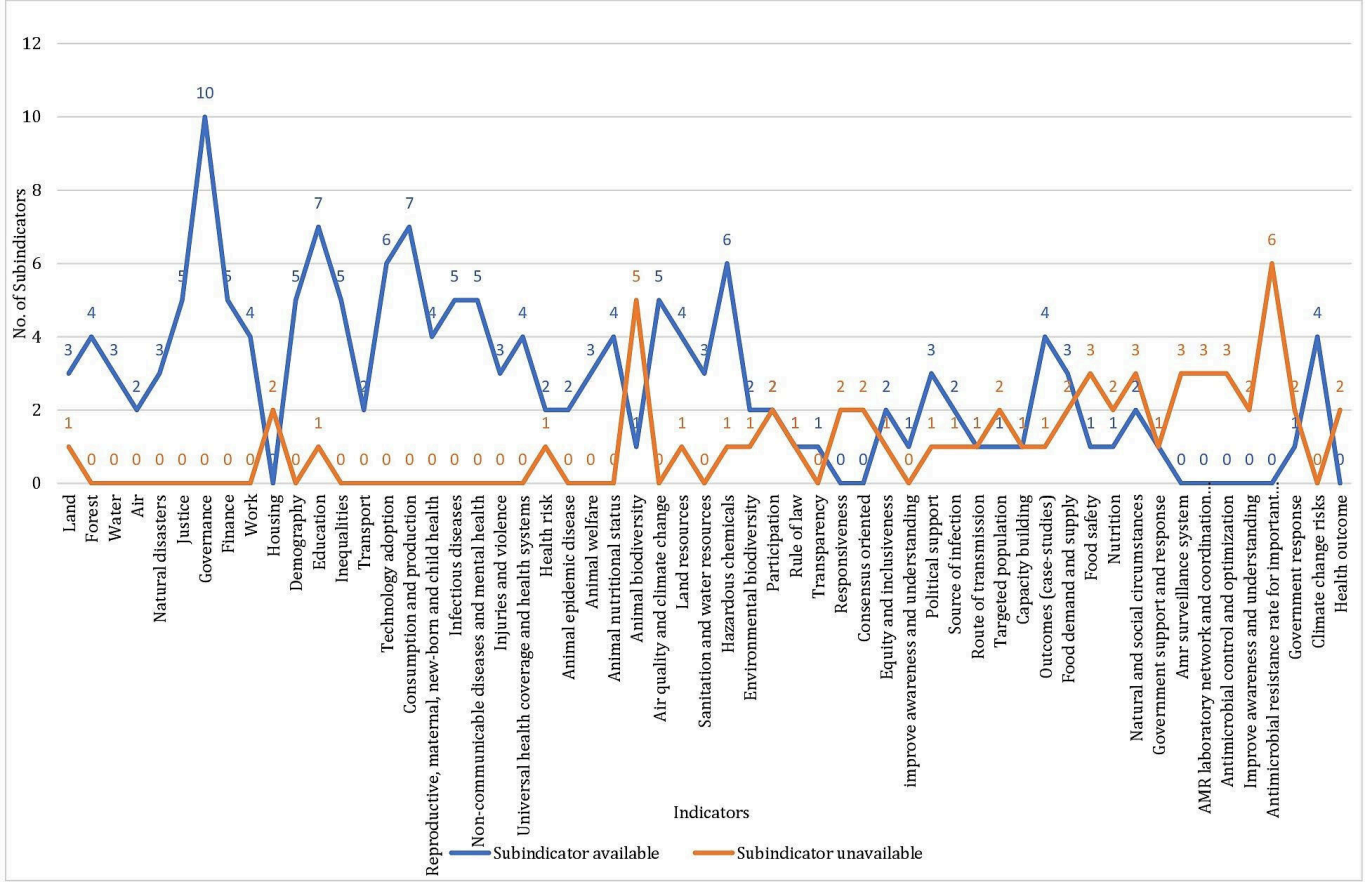
Data availability across indicators. Blue represents the number of subindicators for which the data is available and orange represents the number of subindicators for which the data is unavailable.

Using secondary data sources, a country-specific dataset was developed for India. From this dataset, the indicator values for 156 of the 216 subindicators were gathered, while the remaining 60 subindicators lacked data (Figure 1 and Multimedia Appendix 5). This India-specific dataset reflects data gaps, that is, inconsistency in data availability and areas where there is absence of data requires planned interventions by governance systems. In some cases, due to the absence of historical data, the scores could not be computed.

### Pilot Analysis

**Figure 2.**
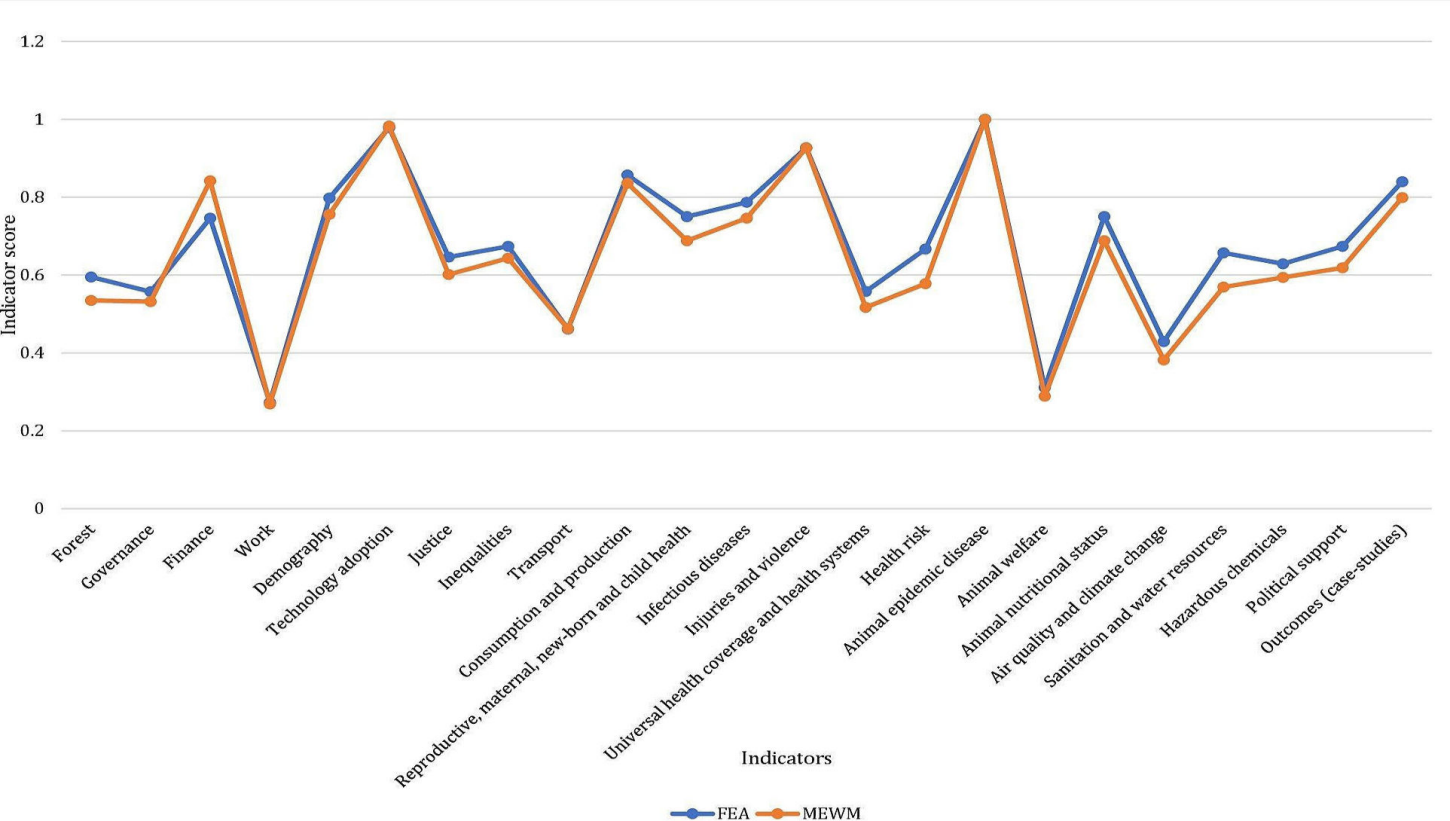
Comparison of indicator scores using FEA and MEWM. FEA: fuzzy extent analysis; MEWM: modified entropy-based weightage method.

**Figure 3.**
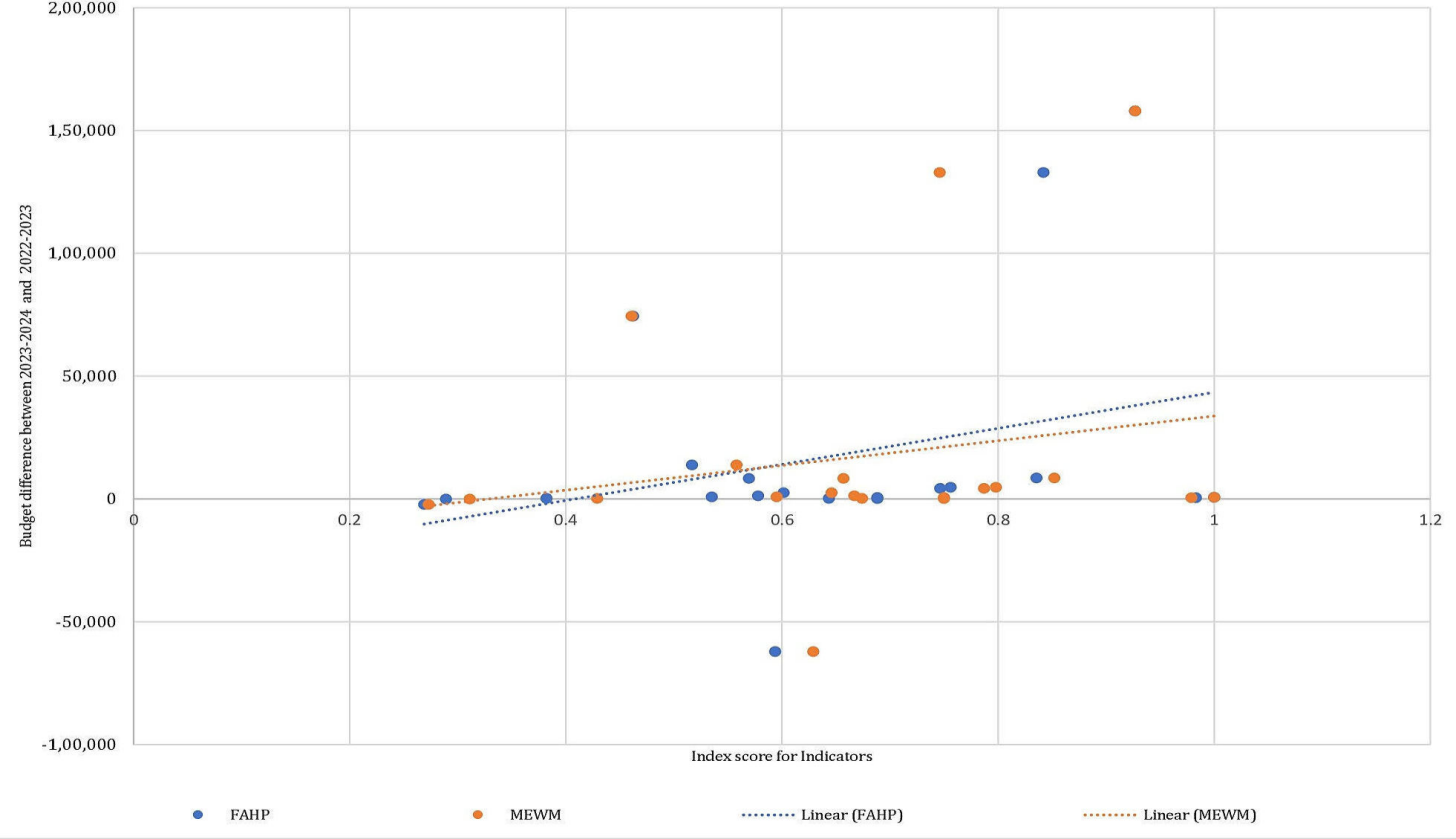
Budgetary differences for the years 2023‐2024 and 2022‐2023 against the indicator scores for consecutive years of budget allocations using the FEA and MEWM. A positive correlation is observed for both methods, indicating that an increase in budget allocation positively impacts the indicator scores. FEA: fuzzy extent analysis; MEWM: modified entropy-based weightage method.

**Figure 4.**
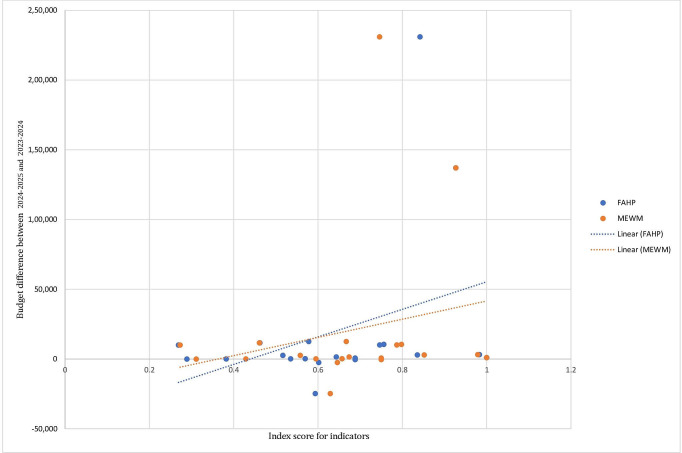
Budgetary differences for the years 2024‐2025 and 2023‐2024 against the indicator scores for consecutive years of budget allocations using the FEA and MEWM. A positive correlation is observed for both methods, indicating that an increase in budget allocation positively impacts the indicator scores. FAHP: fuzzy analytic hierarchy process; MEWM: modified entropy-based weightage method.

Additionally, to evaluate the efficiency of weightage methods, a comparative analysis was conducted between FEA and MEWM. Scores were calculated for 23 indicators for which data for all or most sub-indicators were available (Figure 2). The findings demonstrate that the indicator scores obtained using the two weightage methods, FEA and MEWM for 23 indicators are significantly consistent and thereby render the two methods reliable. Further, to check the OHI calculator’s applicability and utility in the Indian context, a correlation analysis was performed between 20 indicator scores and budget allocations for the years 2022‐2023, 2023‐2024, and 2024‐2025 (Multimedia Appendix 3). The differences in budget allocations for each consecutive year were calculated, aiding in understanding the correlation between the indicator scores and the changes in budget allocations (Figure 3 and Figure 4). Table 1 shows that increases in budget allocations over consecutive years were associated with corresponding increase in indicator scores, and vice versa.

This emphasizes the importance of budgetary allocation in the performance of indicators, thereby impacting the overall performance of *One Health* in India. The OHI calculator has the potential to perform such nuanced correlation analysis; in this case, between budgetary allocation and performance of the indicators through their scores. This can serve as a valuable insight for policymakers and stakeholders alike in prioritizing sectoral interventions related to *One Health*.

**Table 1.** Correlation between the increase in budget and indicator scores.

Difference between years	Indicator scores (FEA)^a^	Indicator scores (MEWM)^b^
2024‐2025 and 2023‐2024	0.353505892	0.22310977
2023‐2024 and 2022‐2023	0.311369994	0.20383558

a FEA: fuzzy extent analysis.

b MEWM: modified entropy-based weightage method.

## Discussion

### Summary

The OHI can be computed at various levels of governance in India, which is constituted of 28 states and 8 Union Territories. Its implementation depends on data availability, such as national, state, district or even block or village/panchayat levels. By calculating these values locally and then aggregating them, an accurate national value can be derived, which highlights demographic variations and provides a more precise measurement. For state-level calculations, population density can serve as a key weighting factor. Collaborative monitoring systems are now recognized as essential for effectively managing pandemics and outbreaks [3]. Thus, developing a OHI calculator can contribute to improved implementation of policies using a participatory *One Health* approach. However, the value of such an index extends beyond compiling data; it has the potential to revolutionize our understanding, management, and actions concerning the complex web of factors that influence global health. The process of calculating a OHI for India sheds light on critical areas requiring systematic interventions, aided by policy decisions, particularly regarding resource allocation and strengthening of governance systems. This framework also promotes a collaborative data federation model to address data gaps—such as incomplete data, lack of timely data, and the absence of appropriate data—and correspondingly advocates for data transparency and ethical data management. Implementing a collaborative data federation model and maintaining consistent data collection will help address these challenges and establish a historical dataset for indexing indicators. This approach encourages intersectoral communication and multistakeholder engagement, garners interest from governance systems, and builds momentum toward improving poorly performing indicators, thereby achieving better *One Health* outcomes for the country. A study focusing on mitigating zoonotic disease risk using the economic approach stated that allocating budget and resources strategically at a higher level secures sufficient funding to manage diseases along the livestock value chain, leading to improvements in human health [16]. As shown in Table 1, increasing the budget over consecutive years has led to higher indicator scores and vice versa, suggesting that budget increases for relevant ministries and departments contribute to improved indicator scores, thereby enhancing the OHI. Addressing the standardization of impact indicators and integrating new field knowledge are also essential.

In this study, we have provided a OHI calculator tool, demonstrating two weighting methods, FEA and MEWM, alongside the India-specific datasets that are accessible and efficient for use by multiple stakeholders such as government functionaries, policymakers, researchers, and institutes of disease surveillance and preparedness. While the scope of this study is to develop a OHI calculator for India, the objective is to provide a reliable, easy-to-use, and efficient tool that could be easily adapted by relevant sectoral stakeholders to compute empirical scores and plan informed and strategic interventions. We have also demonstrated OHI value calculations for India using both methods: the FEA method, which assigns equal weightage in the absence of expert weightage, yielded a score of 46.84, while the score obtained using the MEWM is 42.64. These values demonstrated that OHI calculation values fall within the range reported for the South Asia region, which is 35‐50 as published in 2022, using the GOHI framework [8]. Further, it is also pertinent to conduct expert consultations to review the indicators adopted from the GOHI framework and to develop a list of indicators for India, which may be region-specific and locally adapted.

There are some limitations to be considered regarding the OHI calculator. For instance, the timeline for data availability is inconsistent across subindicators, and any gaps in data may impact the OHI score. Identifying reliable, validated data sources which is crucial for accuracy has been a challenge for many indicators. The selection of experts is a key factor, as their input determines the weightages assigned to different indicators. Any bias from the experts or the absence of a representative sample may result in a skewed OHI score. To avoid regional bias, it is crucial to ensure diverse representation from across the country for balanced input.

### Conclusion

The OHI calculator identifies key areas that require concerted action from *One Health*-centric stakeholders. During its development, data gaps and deficiencies for crucial *One Health* indicators were identified, suggesting that data federation and open data access from governance systems and research organizations working in the public interest should emerge as actionable agendas for collaborative efforts. In this context, the methodology and framework for calculating a single OHI value require multisectoral experts to come together to improve the OHI for India by recognizing the need for sectoral data. This in turn, leads to identifying disparities, targeting interventions, monitoring health trends, and implementing other strategic efforts aligned toward the health equity paradigm. Additionally, the importance of budgetary allocation for improving indicator scores that contribute to OHI, serves as a reminder to policymakers about the value of empirical and evidence-based decision making. The process of calculating an empirical OHI value demands consistent public engagement and awareness of the interconnected nature of the *One Health* approach. Ultimately, this leads to better preparedness for handling future pandemics, improved quality of life, and progress toward achieving the sustainable development goals.

## Supplementary material

10.2196/65039 Multimedia Appendix 1 Global One Health Index (GOHI) framework list.

10.2196/65039 Multimedia Appendix 2 India dataset.

10.2196/65039 Multimedia Appendix 3 Budgetary allocation sheet vs. indicator scores.

10.2196/65039 Multimedia Appendix 4 Sample proforma for expert rating.

10.2196/65039 Multimedia Appendix 5 List of subindicators for which data is unavailable.

## Abbreviations:

AMR: Antimicrobial resistance
FEA: fuzzy extent analysis
GOHI: Global One Health index
MEWM: Modified Entropy-based Weightage method
OHI: One Health Index
